# Uncertainty in climate change impact studies for irrigated maize cropping systems in southern Spain

**DOI:** 10.1038/s41598-022-08056-9

**Published:** 2022-03-08

**Authors:** Bahareh Kamali, Ignacio J. Lorite, Heidi A. Webber, Ehsan Eyshi Rezaei, Clara Gabaldon-Leal, Claas Nendel, Stefan Siebert, Juan Miguel Ramirez-Cuesta, Frank Ewert, Jonathan J. Ojeda

**Affiliations:** 1 Leibniz Centre for Agricultural Landscape Research, Eberswalder Straße 84, 15374 Müncheberg, Germany; 2 Agricultural Faculty, Institute for Crop Science and Resource Conservation (INRES), University of Bonn (UNIBONN), Katzenburgweg 5, 53115 Bonn, Germany; 3 IFAPA - “Alameda del Obispo”, Avda. Menéndez Pidal s/n., 14004 Córdoba, Spain; 4 Department of Crop Sciences, University of Göttingen, Von-Siebold-Strasse 8, 37075 Göttingen, Germany; 5 Institute of Biochemistry and Biology, University of Potsdam, Am Mühlenberg 3, 14476 Potsdam (Golm), Germany; 6 Dpto. Riego, Centro de Edafología y Biología Aplicada del Segura (CEBAS-CSIC), P.O. Box 164, 30100 Murcia, Spain; 7 Tasmanian Institute of Agriculture, University of Tasmania, Sandy Bay Campus, Hobart, TAS 7005 Australia; 8 Queensland Alliance for Agriculture and Food Innovation, The University of Queensland, Brisbane, QLD 4072 Australia

**Keywords:** Climate sciences, Environmental sciences

## Abstract

This study investigates the main drivers of uncertainties in simulated irrigated maize yield under historical conditions as well as scenarios of increased temperatures and altered irrigation water availability. Using APSIM, MONICA, and SIMPLACE crop models, we quantified the relative contributions of three irrigation water allocation strategies, three sowing dates, and three maize cultivars to the uncertainty in simulated yields. The water allocation strategies were derived from historical records of farmer’s allocation patterns in drip-irrigation scheme of the Genil-Cabra region, Spain (2014–2017). By considering combinations of allocation strategies, the adjusted *R*^2^ values (showing the degree of agreement between simulated and observed yields) increased by 29% compared to unrealistic assumptions of considering only near optimal or deficit irrigation scheduling. The factor decomposition analysis based on historic climate showed that irrigation strategies was the main driver of uncertainty in simulated yields (66%). However, under temperature increase scenarios, the contribution of crop model and cultivar choice to uncertainty in simulated yields were as important as irrigation strategy. This was partially due to different model structure in processes related to the temperature responses. Our study calls for including information on irrigation strategies conducted by farmers to reduce the uncertainty in simulated yields at field scale.

## Introduction

Irrigated agriculture constitutes 24% of the global crop area and accounts for over 40% of world’s food production^1^. It generally leads to higher and more stable yield, as additional water buffers yield-limiting factors such as drought^2,3^ and high temperatures^4,5^. In recent decades, irrigation development has played a key role in overcoming the dual challenges of agriculture, namely meeting increasing food demand^6^ and ensuring production in the face of climate change^4^. Irrigated agriculture is considered as an important adaptation strategy not only to drought, but also to the increased number and severity of heat waves projected under climate change^2,7^. Irrigation acts to buffer the effect of extreme temperature on crop physiology through cooling of the plant’s surface when water is being transpired^8^. Despite its importance for supporting food production, irrigated agriculture is associated with various environmental challenges, of which limited water resources are a primary concern. Competition between sectors^8,9^ for already limited water resource availability^10,11^ is projected to increase under climate change^12,13^. Therefore, it is imperative to have robust assessments on the potential of irrigation as a climate change adaptation and support appropriate allocation of scarce water resources^14^.

Process-based crop models (hereafter crop models) enable projecting crop responses to climate change for a range of management adaptation options, such as irrigation, sowing date, or cultivar choice^15,16^. Currently, most large-scale climate change impact studies simulate crop growth under full irrigation and rainfed conditions and aggregate the yields based on the relative share of cropping area in each system^17^. However, to date, such approaches largely fail to capture the inter-annual yield variability in irrigated systems^18^. Possible explanations include the lack of information on water availability at the farm gate^19,20^ as well as lack of information on farmer’s water management in response to water shortage^21,22^. A third possible explanation relates to uncertainties in growth processes simulated in crop models^23,24^ related to the role of transpiration cooling under irrigation^14,25^. Beyond these, uncertainties in cultivars, sowing dates, fertilization, and crop protection exist, as in rainfed production systems. These sources of uncertainties challenge not only reproducing historical simulations but also creating uncertainties in projecting future climate change scenarios^9,26^.

**Figure 1:**
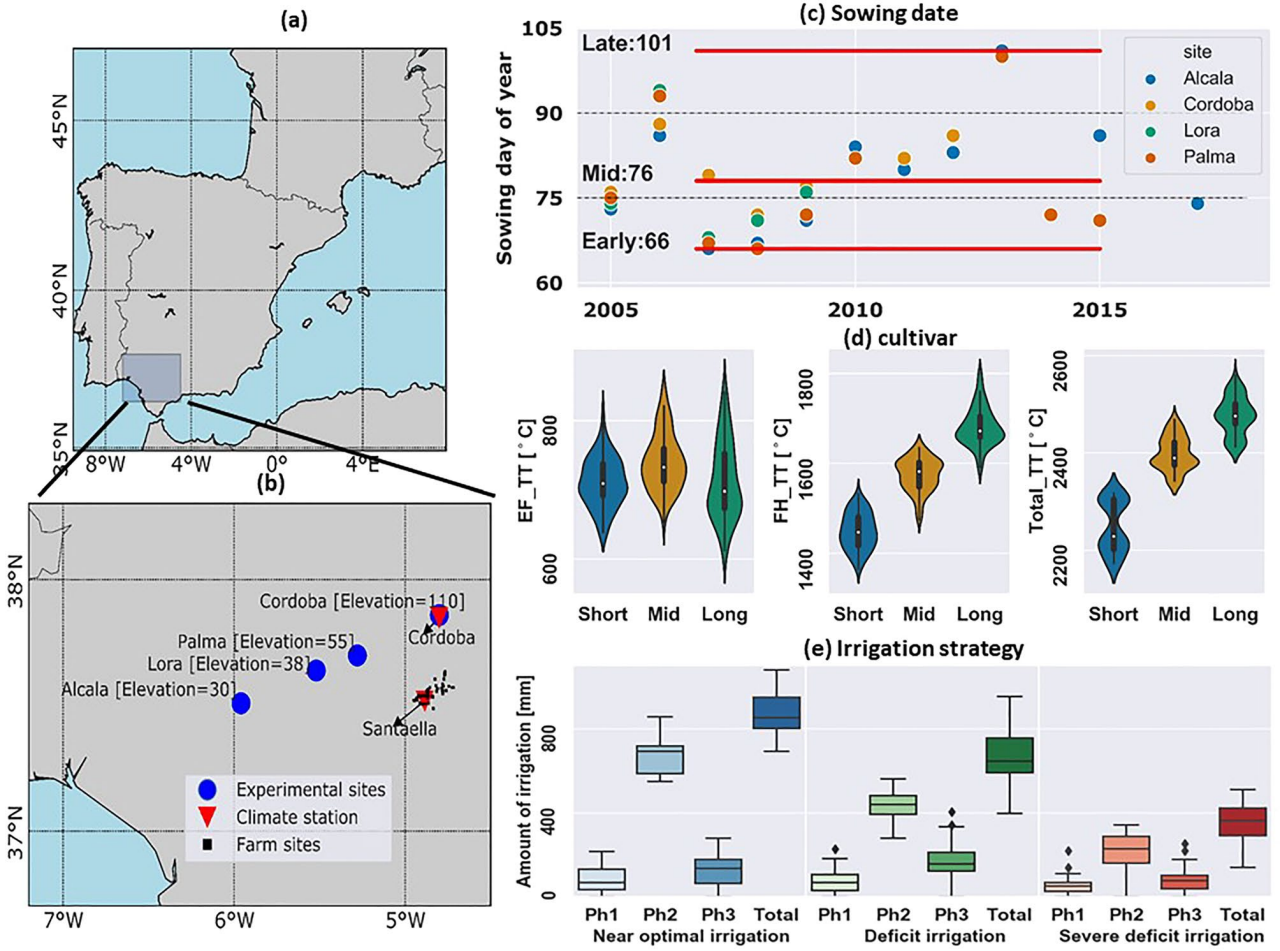
Left panels: (**a**) Location of the Andalusia region in Spain; (**b**) location of the four experimental sites (blue circles), Cordoba and Santaella climate stations (red triangles), and 118 farm sites (black points). Right panels: Classification of factors; (**c**) Sowing date: early, mid, and late sowing days of year; (**d**) Cultivar: short, mid, and long cultivars calculated based on three thermal time sums (EF_TT: sum of thermal time from emergence to flowering, FH_TT: sum of thermal time from flowering to harvest, Total_TT: sum of thermal time from emergence to harvest); (**e**) Irrigation strategy: Near optimal, Deficit, and Severe deficit irrigation strategies. The boxplots in panel (**e**) show the amount of water applied during the three phases of maize growth (from sowing to May (Ph1), from June to July (Ph2), and from August to harvest (Ph3)) and during the total crop growing season (Total). The data used to produce this figure has been obtained from farm-level and experimental field level^22,27^. The map has been generated with using Python 3.7.4

Identifying the relative importance of these sources of uncertainty would aid in prioritizing which model improvements or additional data are more critical for assessing climate impacts on irrigated systems. A better understanding of the importance of various sources of uncertainties in simulating irrigated crop yields in large-scale studies will improve climate change impact assessments, supporting the design of adapted cropping systems. For this purpose, we investigated the relative uncertainty stemming from each of the following factors: (1) irrigation water allocation strategy (hereafter irrigation strategy), (2) sowing dates, (3) cultivar maturity group (i.e. short, mid, or long cultivar), (4) crop models for various scenarios of temperature increase and altered irrigation water availability. We investigated this for irrigated maize fields in the Genil-Cabra Irrigation Scheme (GCIS) in the middle-low section of the Guadalquivir River basin, Andalusia, southern Spain, where ~ 80% of the available water resources of the basin are used for irrigation (Fig. 1a). Specifically, we explore three questions.

1. Which combination of plausible irrigation strategies best explains the inter-annual irrigated yield variability in historical statistics for Mediterranean regions under semi-arid conditions?
2. Which factor(s) (irrigation strategy, sowing date, cultivar, or crop model) contribute(s) most to uncertainty in simulated yield?
3. How do the relative contribution of these factors vary under different climate warming and water availability scenarios?

## Data and methods

### Data overview

Three levels of data were available for the study area and used for our analyses:

1. *Experimental field-level* this dataset included crop phenological information (days of sowing, emergence, flowering, and physiological maturity) and maize grain yields (hereafter yield) for over 70 cultivars (Figs. S1 and S2) at four experimental sites, namely: Alcala, Palma del Rio, Lora, and Cordoba (Fig. 1b) during 2005–2017^27^ under non-water stress and non-nitrogen stress conditions. We used this dataset to calibrate the crop models.
2. *Farm-level* This data included the amount of irrigation supply with drip systems^22^ at 118 farms located in a representative irrigation scheme in GCIS with farm sizes varying between 0.5 and 20 ha during 2014-2017 (Fig. 1b). The farm-level irrigation data were collected during three periods within the maize growing season: from sowing to May (Ph1), from June to July (Ph2), and from August to harvest (Ph3). Farmers applied the highest amount of irrigation in June and July as the hottest and driest time of the year, coinciding with maximum crop coverage. This dataset was used to derive irrigation strategies applied in the region.
3. *Regional-level* This data included average irrigation water availability (for all crops) in Andalusia region during 1990–2018 (Fig. S1a) and were obtained from the Spain Ministry of agriculture^28,29^. The statistical maize yields at the Nomenclature of Territorial Units for Statistics (NUTS2) level were obtained from CAPRI (Comparative Analysis of PRotein-protein Interaction) database (Fig. S1b). This dataset was used to evaluate the impact of adding information from different irrigation strategies and irrigation water availability on improving simulation of maize yields. An increasing trend in maize yields of Andalusia was detected for 2000–2018, which might be due to improvements in irrigation efficiency, cultivar, or crop technology improvement. To remove the impact of such factors, we de-trended yields during this period.

### Weather

The daily weather variables, including precipitation, maximum temperature, minimum temperature, solar radiation, and wind speed in the Santaella station (located within the GCIS) during 1979–2018 were used for all three levels of datasets with exception of Cordoba site from experimental field-level for which we used the weather variables from Cordoba station (Fig. 1b).

### Soil

The predominant soil in experiment and farm sites is a clay loam with 31% clay, 33% sand, and 36% silt. The soil types are Chromic Haploxererts (35%) and Typic Xerorthent (35%)^30^. The reported values for soil water content at field capacity and permanent wilting point are 0.33 mm^3^ mm^−3^ and 0.18 mm^3^ mm^−3^ (profile average), respectively (see Table S1 for soil profile characteristics at the layer level up to 2000 mm depth)^21^. As initial soil moisture, soil water content at the beginning of the crop growing season, was not available, we assumed in the model simulations that at the end of the previous growing season (e.g. September 2017) soil moisture was at the lowest level. The water balance was then simulated from the end of the previous season to the beginning of the given season (e.g. March 2018).

## Classification of management factors for uncertainty analysis

### Sowing dates

Using sowing dates recorded at the four experimental sites, early, mid, and late sowing date classes were defined as the earliest, middle, and latest days of year recorded during 2005–2015 (Fig. S2), which were 66, 76, 101, respectively (Fig. 1c).

### Cultivars

We used the recorded phenological dates for a range of cultivars at the four experimental sites and assumed a base temperature of 8 °C for maize^31^. Next, we calculated the thermal time (having assumed no photoperiod sensitivity) from emergence to flowering (EF_TT) and from flowering to harvest (FH_TT). Assigning EF_TT and FH_TT as the features, we carried out the *k-mean* clustering method^32^ to classify cultivars into three main classes of short, mid, and long. The EF_TT values for short, mid, and long cultivars were 701 °C, 732 °C, and 691 °C, respectively (Fig. 1d). For short, mid, and long cultivars, the FH_TT values were 1449 °C, 1577 °C, and 1682 °C, respectively (Fig. 1d).

### Irrigation strategies

The *k-mean* clustering method was also applied to stratify irrigation strategies for model simulation. The amounts of water applied by farmers during three phases of growing seasons (Ph1, Ph2, and Ph3) were taken as the input features for the clustering method. The farm-level data were available during the period 2014–2017 with about 58% of data belonging to 2014. Therefore, to reproduce the year-to-year variation of the irrigation strategies during the studied period (1990–2018), we assigned the extracted features of each derived class (strategy) from *k-means* to 2014 as the reference year. We then used the ratio of yearly water availability at regional-level to the value in 2014. The ratios were then used to reproduce yearly water allocations at farm-level. As follows, we derived three clusters, i.e., irrigation strategies from our clustering method which were defined as (Fig. 1e):

1. *Near optimal* Maize gets most available water possible. This scenario happens in wet years with high levels of water availability or in dry years when available water resources are concentrated in a reduced area for maize cultivation. The total amount of irrigation in this strategy is 830 mm year^−1^.
2. *Deficit* Maize gets normal (average) share of irrigation allocation. The total amount of irrigation in this strategy is 620 mm year^−1^.
3. *Severe deficit* Maize gets much less water in a dry year due to the lack of available resources for irrigation or priority of available resources is given to other crops. The total amount of irrigation in this strategy is 370 mm year^−1^.

For all strategies, an average irrigation efficiency (the ratio of the amount of water consumed by the crop to the amount of water supplied through irrigation) of 0.85 was considered for both the experiments and farms which is also according to the study of Santos, et al.^33^ and adjusted by agronomists and regional expert consultation.

### Crop models

Three process-based crop models were used in the study to simulate maize growth and development at a daily step: APSIM^34^ (Agricultural Production Systems sIMulator), MONICA^35^ (Model for Nitrogen and Carbon dynamics in Agro-ecosystems), and a solution in the SIMPLACE framework, referred to as SIMPLACE-Lintul-5^8^. The main features of the models in terms of how they simulate water demand and uptake, as well as the effects of water and heat stress were presented in Appendix. 1 and Tables S2 and S3. Simulations were conducted assuming no nitrogen limitation reflecting practices of farmers in the region. The three models share three phenological phases in common: sowing to emergence, emergence to flowering, and flowering to maturity, though APSIM and MONICA each additionally consider other intermediate stages (Table S3). The common phenological phasees enabled us to calculate thermal time at these three phases which was assimilated them into each model to assure that all crop models followed the same pattern in terms of phenological development.

The models were calibrated at the experimental field level. We calibrated models based on the yield data available for a variety of cultivar (Fig. S3) and flowering dates recorded in the four experimental sites. To analyze model accuracy, observed and simulated yield values were compared using the Root Mean Square Error (*RMSE*) and the relative RMSE (*rRMSE*).

### Plausible past combinations of irrigation strategies

**Figure 2:**
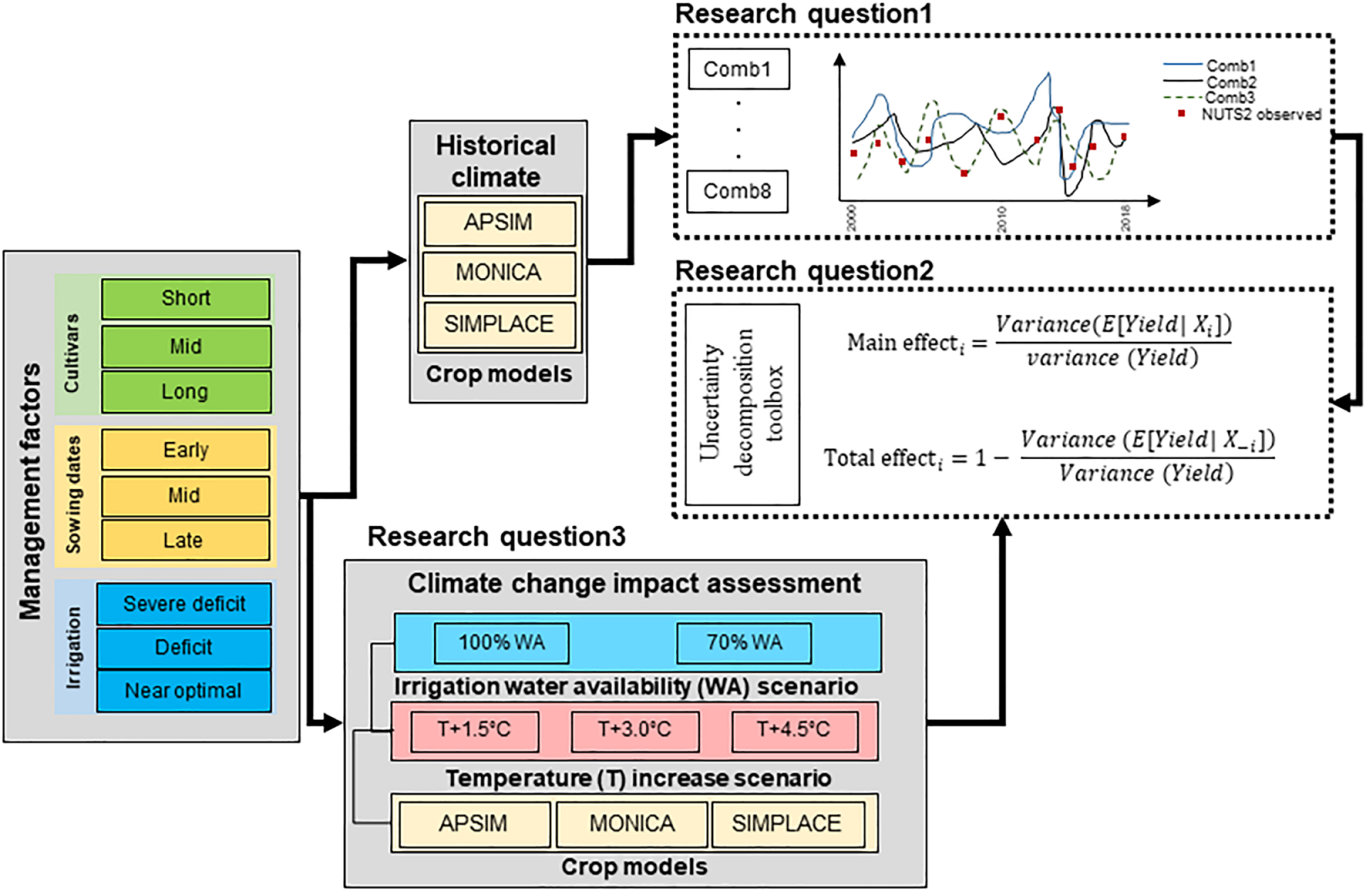
Schematic representation of methodology applied to answer three research questions definded for this study (see Introduction section).

The three calibrated models were applied to simulate maize yield at regional-level (Andalusia in southern Spain) using different combinations of irrigation strategy, sowing date, and cultivar choice (Fig. 2). We determined simulated yields obtained from eight different linear combinations of three irrigation strategies (Comb1-8 in Table 1) and nd compared with historical yields recorded for NUTS2-regions in Andalusia region (southern Spain) during 1990–2018. In the first level, we purely looked at the influence of irrigation strategy and excluded the contribution of other factors (i.e. sowing date, cultivar, and crop model) to yield variability. To do so, we built the regression model based on one (out of three) type of sowing date, one type of cultivar, and one crop model at a time (e.g. mid sowing date, mid cultivar and one of the crop model). In the next levels, we explored the contribution of other three factors in capturing yield variability. We added the influence of the other three factors to these eight combinations successively in three levels (Comb1-8). That is, in the second level we added the variability from sowing date to irrigation strategy (Irrigation strategy + sowing date) and re-built Comb1-8 considering the average yields simulated under all three types of sowing date. Similarly, we considered the average yields simulated under three types of cultivar in the third level (Irrigation strategy + sowing date + Cultivar), and the average yields simulated under three crop models in the fourth level (Irrigation strategy + Sowing date + Cultivar + Crop model).

**Table 1:** Linear combination of three different irrigation strategies.

Combination name	Linear combination of different irrigation strategies
$Comb1$	$simulated \,yield = Yield_{Severe\, deficit}$
$Comb2$	$simulated \,yield = Yield_{Deficit}$
$Comb3$	$simulated \,yield = Yield_{Near \,optimal}$
$Comb4$	$simulated \,yield = a \times Yield_{Severe\, deficit} + b \times Yield_{Deficit}$
$Comb5$	$simulated\, yield = a \times Yield_{Severe \,deficit} + b \times Yield_{Near\, optimal}$
$Comb6$	$simulated \,yield = a \times Yield_{Deficit} + b \times Yield_{Near\, optimal}$
$Comb7$	$simulated\, yield = (Yield_{Severe \,deficit} + Yield_{Deficit} + Yield_{Near \,optimal})/3$
$Comb8$	$simulated \,yield = a \times Yield_{Severe \,deficit} + b \times Yield_{Deficit} + c \times Yield_{Near\, optimal}$

We repeated the above sequential concepts to quantify the contribution of sowing date (or cultivar) for capturing yield variability in the absence of variability from irrigation strategy. To do so, we obtained simulated yields from eight combinations of sowing dates (or cultivars) and quantified their performance in terms of capturing yield variability. Table S4 illustrates the list of combinations built using different combinations of sowing date (or cultivar).

The degree of agreement between recorded yields in regional-NUTS2 and simulated yields was evaluated using the adjusted coefficient of determination (adjusted *R*^2^)^36^. The adjusted R^2^ is used to compare models with different number of explanatory variables and to identify the best model by minimizing the variability of the dependent variables with respect to the independent variables^36^. As one of the most common and advanced approaches^37^, we reported two additional criteria to compare the degree of improvement in the regression model by adding more factors: (1) the *p-value* shows the statistical significance and reflects the difference in central tendency and the number of observations, and 2) *eta squared*^38^ as a measure of effect size expresses the expected difference or ratio in the outcome between two interventions^39^. Recent studies have shown that both criteria are essential to understand the full impact of different factors in building models. Effect sizes measures the magnitude of differences, whereas statistical significance examines whether the findings are likely to be due to chance^37^.

### Simulation experiment and uncertainty decomposition

The decomposition analysis was conducted to assess how much uncertainty of irrigation strategy, sowing dates, cultivars, and crop models contributed to simulated yields (Fig. 2). We also explored the decomposition analysis under historical climate conditions, three scenarios of temperature (T) increase ([Historic T] + 1.5 °C, [Historic T] + 3.0 °C and [Historic T] + 4.5 °C), and one scenario of shortage in water availability i.e., 70% water availability. Simulating conditions with reduced water availability are representative of cases when farmers are constrained by the amount of allocated irrigation water or when irrigation of other crops are prioritized by farmers. Although scenarios of reduced water availability deviates from near-optimal irrigation strategies, but model results comparison on such hypothetical scenarios enables us to simulate the impact under double constraints of water allocation and high temperature on yield production. We analyzed the contribution of each factor on the basis of the variability of yields over time. The drivers of yield variability during years with water availability below average (low water availability years) were compared with years when water availability was above average (high water availability years) (Fig. 2).

To determine the contribution of each factor to the total crop yield variability, the variance-based sensitivity indices were computed^40^. According to this method, the variance of the crop model output is decomposed into fractions which can be attributed to various factors. Variance-based measures of sensitivity are attractive because they measure sensitivity across the whole input space and therefore deal with nonlinear responses. They measure the sensitivity of each factor independently and also quantify the effect of interactions in non-additive systems. Two indices, namely main effects, and total effects used to disentangle the variance caused by one factor from the variance caused by the interaction are:

1 $${\text{Main effect}}_{i} = \frac{{Variance(E[Yield|{ }X_{i} ])}}{{variance \left( {Yield} \right)}}$$

and

2 $${\text{Total effect}}_{i} = 1 - { }\frac{{Variance (E[Yield|{ }X_{ - i} ])}}{{Variance \left( {Yield} \right)}}$$

where $E[Yield|X_{i} ]$ denotes the expected value of maize yield across all factors *X*_*i*_, while $E[Yield|X_{ - i} ]$ is the expected value of maize yield across all factors except *X*_*i*_. The main effect index measures the contribution of one varying factor X alone; however, it is averaged over variations in other factors. The main effect is standardized by the total variance which enables to obtain fractional contribution. Therefore, the sum of the main effect values obtained for each factor is always smaller than one. On the other hand, the total effect determines the contribution of various factors to the total variability in maize yield including all variance caused by its interactions of any order. The total effect is the sum of the main effect and all the higher-order sensitivity indices involving that parameter; therefore, the sum of total effect values generally will be more than one^41^. The variance-based analysis for this paper has been conducted using Python 3.7.4. The software has been also used to generate all figures presented in this paper.

## Results

### Maize grain yield variability in the historical period

**Figure 3:**
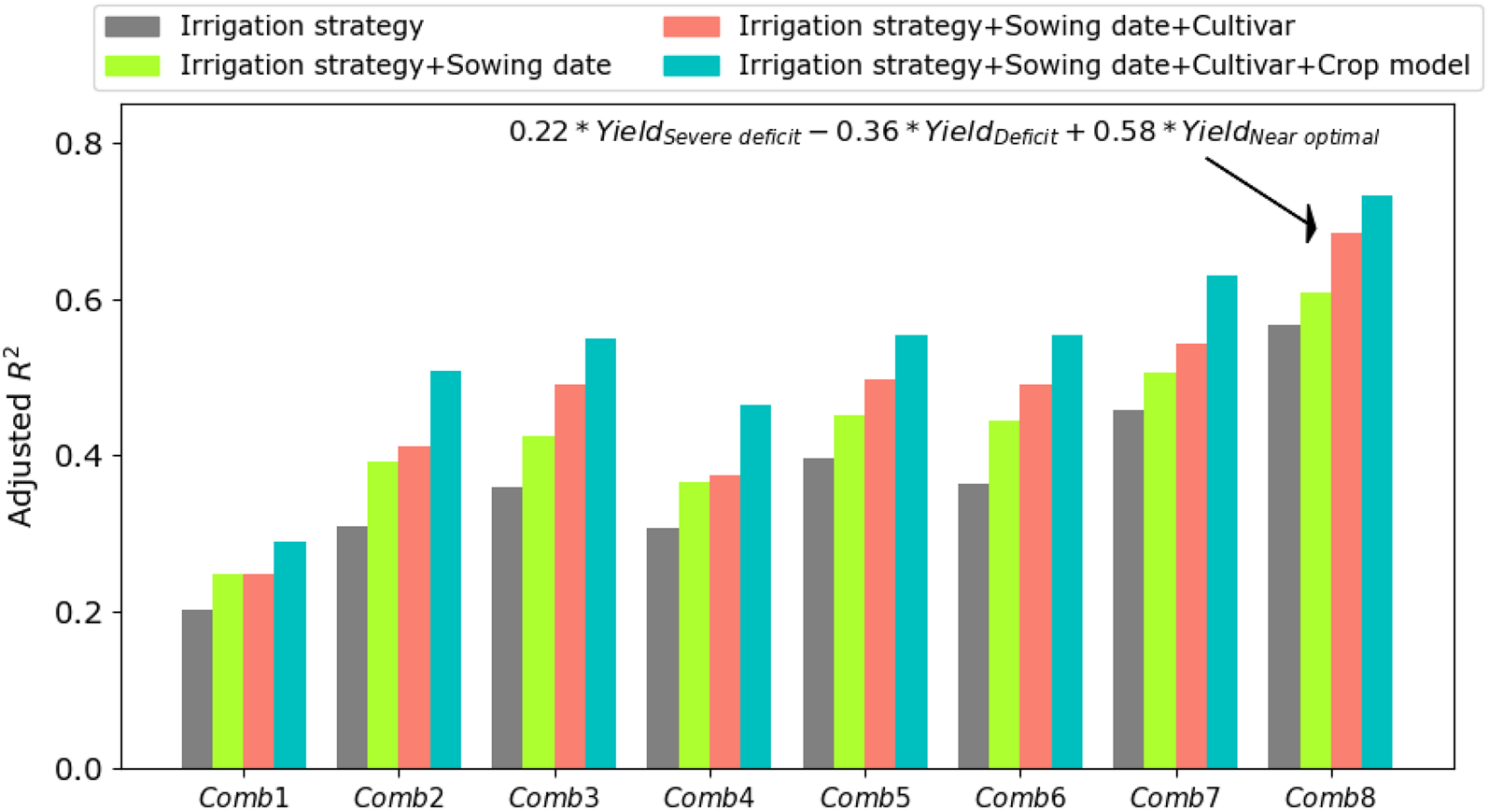
The adjusted coefficient of determination (*R*^2^) indicating the degree of agreement between recorded maize grain yield at regional-level in Andalusia and simulated maize yield calculated based on eight different combinations of irrigation strategies (Comb1-8 in Table 1) and other factors as sowing date, cultivar, and crop model during 1990–2018.

The crop models were used to simulate regional yield under different combination of irrigation strategies, sowing date, and cultivar. Comparing simulated yields from the eight combinations of irrigation strategies (Comb1-8) with regional-NUTS2 observed yields indicated that simulated yields obtained from combination of three irrigation strategies (Comb7 and Comb8) had higher adjusted *R*^2^ values than those yield simulations consisting of one individual strategy (Comb1-3) (see grey bar in Fig. 3). The near optimal irrigation strategy (Comb3) is more frequent for maize than severe deficit irrigation strategy of (Comb1) and therefore the adjusted R^2^ of 0.36 for Comb3 outperformed Comb1 (severe deficit strategy) with value of 0.20 (grey bars in Fig. 3). Despite this outperformance for Comb3, the adjusted R^2^ value further increased to 0.56 in Comb8 which reveals the critical role of all irrigation strategies together for capturing yields variability in our simulations. The adjusted R^2^ further increased from 0.56 to 0.73, when variability from other factors (sowing date + cultivar + crop model) were included in Comb8 (Fig. 3). For almost all regression models, the *p-values* were below 0.01 (less than 0.01 significance level) showing that the regression model provides a satisfactory fit to the data (Table S5). Comparing the *eta squared* values (indicator of effect size) of different combinations showed values of 0.81–0.87 for Comb8 (including three irrigation strategies) which was larger than values for Comb1-7 with *eta squared* < 0.4 (Table S5. This confirmed that irrigation strategy carried the highest level of variance in simulated yields.

Moreover, regional level observed yields were compared with simulated yields obtained from different combination of sowing dates (Fig. S4a). The results showed that combination based on all three sowing dates ($a \times Yield_{Early} + b \times Yield_{Mid} + c \times Yield_{Late}$) outperformed those simulated yields based on one type of sowing date ($Yield_{Early}$, $Yield_{Mid}$, or $Yield_{Late}$) (grey bar in Fig. S4a). However, remarkable improvement in simulated yields was achieved when we included the variability from different irrigation strategies (blue bar in Fig. S4a) compared to combinations based on only sowing dates (grey bar in Fig. S4a). Similarly, we noticed improved performance when we added variability from different irrigation strategies to different combinations of cultivars ($a \times Yield_{Short} + b \times Yield_{Mid} + c \times Yield_{Long}$) (Fig. S4b). It is worth mentioning that all three crop models were calibrated and their performances were evaluated in terms of simulating maize yields and days of flowering in four experimental sites at cultivar level (Fig. S5). The *RMSE* values for yield calibration varied between 0.87 t ha^−1^ for MONICA as the best performing model and 1.48 t ha^−1^ for APSIM as the worst performance. For flowering date assessment, the three crop models showed approximately similar performance in terms of simulating days of flowering (*RMSE* of 1.75, 1.97, and 2.45 days for MONICA, SIMPLACE, and APSIM, respectively).

### Sources of uncertainty in simulated historical grain yields

**Figure 4:**
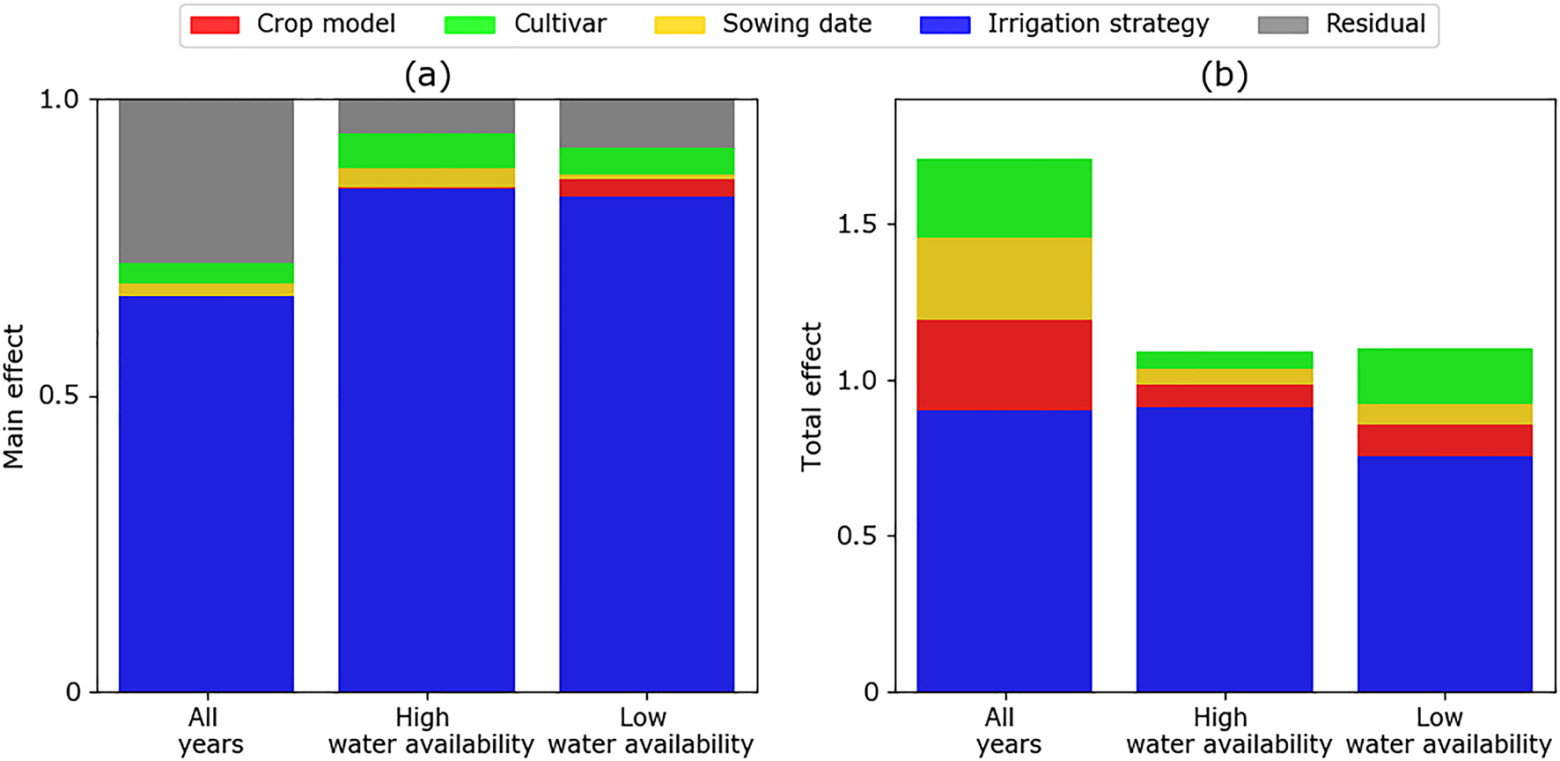
The main effect (**a**) and total effect (**b**) of sensitivity indices as estimated with data from a historical period (1990–2018) presented for all-years, years with low water availability, and years with high water availability.

The results of uncertainty analysis conducted for all years as well as the low water availability and high water availability years indicated that irrigation strategy explained the greatest amount of variation in simulated yields compared to the other factors (Fig. 4). The values of the main effect for irrigation strategy were above 0.65 under all water availability conditions (0.66 for all years, 0.85 for years with high water availability, and 0.83 for years with low water availability). The contributions of sowing date, cultivar, or crop model factors were less than 3% under all water availability conditions i.e. the main effect values were less than 0.03 (Fig. 4). However, phenological features here referred to as cultivar (compared with sowing date and crop model) showed slightly higher contribution to yield variability during years with high water availability (main effect = 0.06) compared to all years (main effect = 0.035). The values for the total effect were higher for irrigation strategy than other factors emphasizing the high interaction of this factor with others.

**Figure 5:**
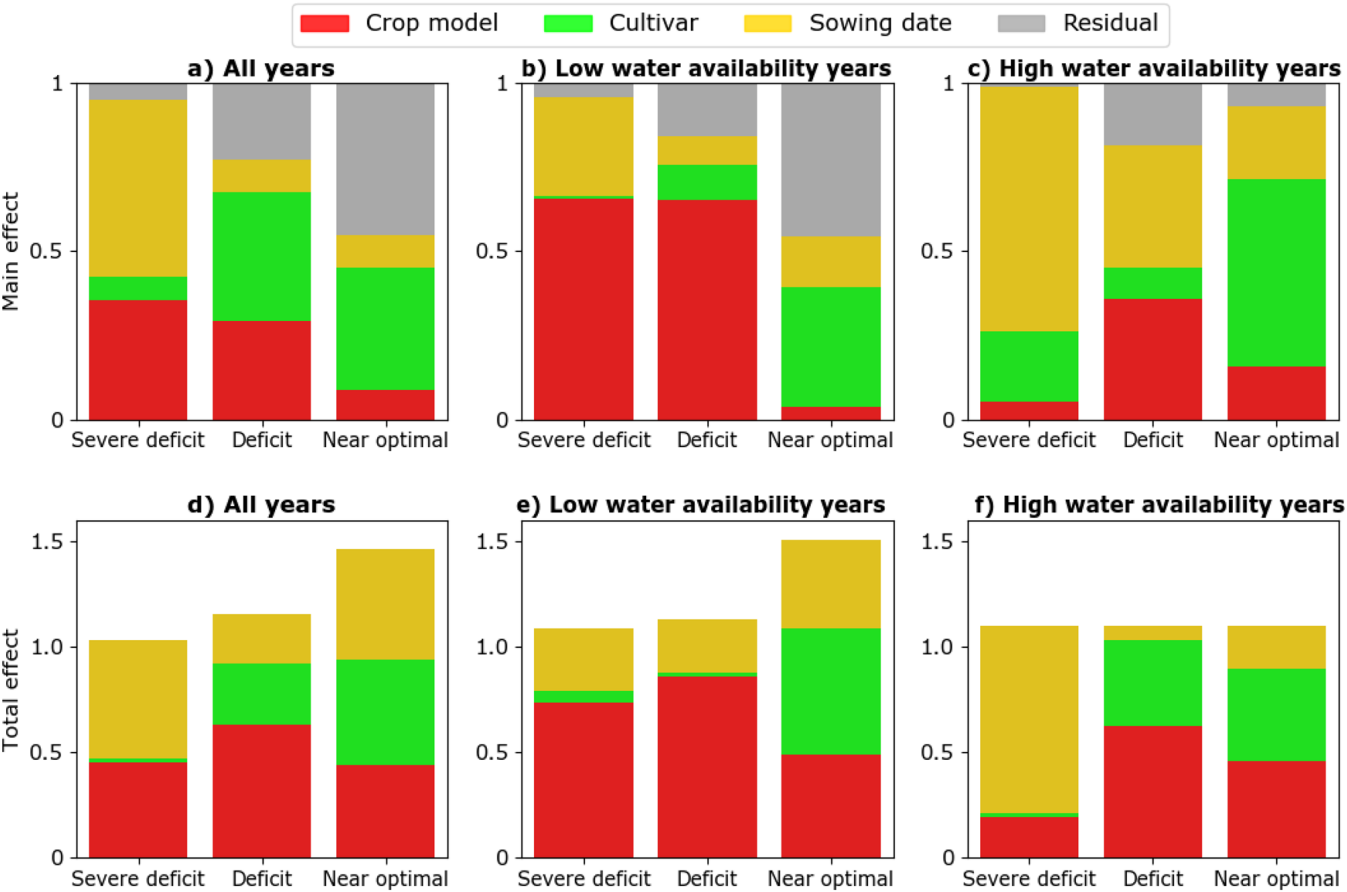
The main effect (**a**–**c**) and total effect (**d**–**f**) of sowing date, cultivar, crop model under three irrigation strategies (Severe deficit, Deficit, Near optimal) explaining variability in simulated yields during all years (**a**,**d**), low water availability years (**b**,**e**), and high water availability years (**c**,**f**).

The decomposition of factors was repeated under each irrigation strategy (Fig. 5). The results for all years showed that the relative importance of factors in explaining simulated yield variation differed depending on the irrigation strategy (Fig. 5a). Under the Near optimal and Deficit irrigation strategies, cultivar explained the most variation followed by crop model, whereas sowing date explained the most variation under the Severe deficit irrigation strategy (Fig. 5a). The decopmosition analysis indicates how factors with the highest contribution to variability in simulated yields differed between low water availability years (Fig. 5b) than from high water availability years (Fig. 5c). In low water availability years, crop models contributed the largest share to yield variation in simulated maize yields (over 50%) for both the Deficit and Severe deficit irrigation strategies. In contrast, during years with high water availability, sowing date explained most of the variation. The results for the total effect showed higher interactions of sowing date and crop model under all irrigation strategies and for both low and high water availability cases (Fig. 5d–f). The interaction of cultivar with other factors increased under the Near optimal irrigation strategy.

### Factor importance under increased temperatures and reduced water availability

**Figure 6:**
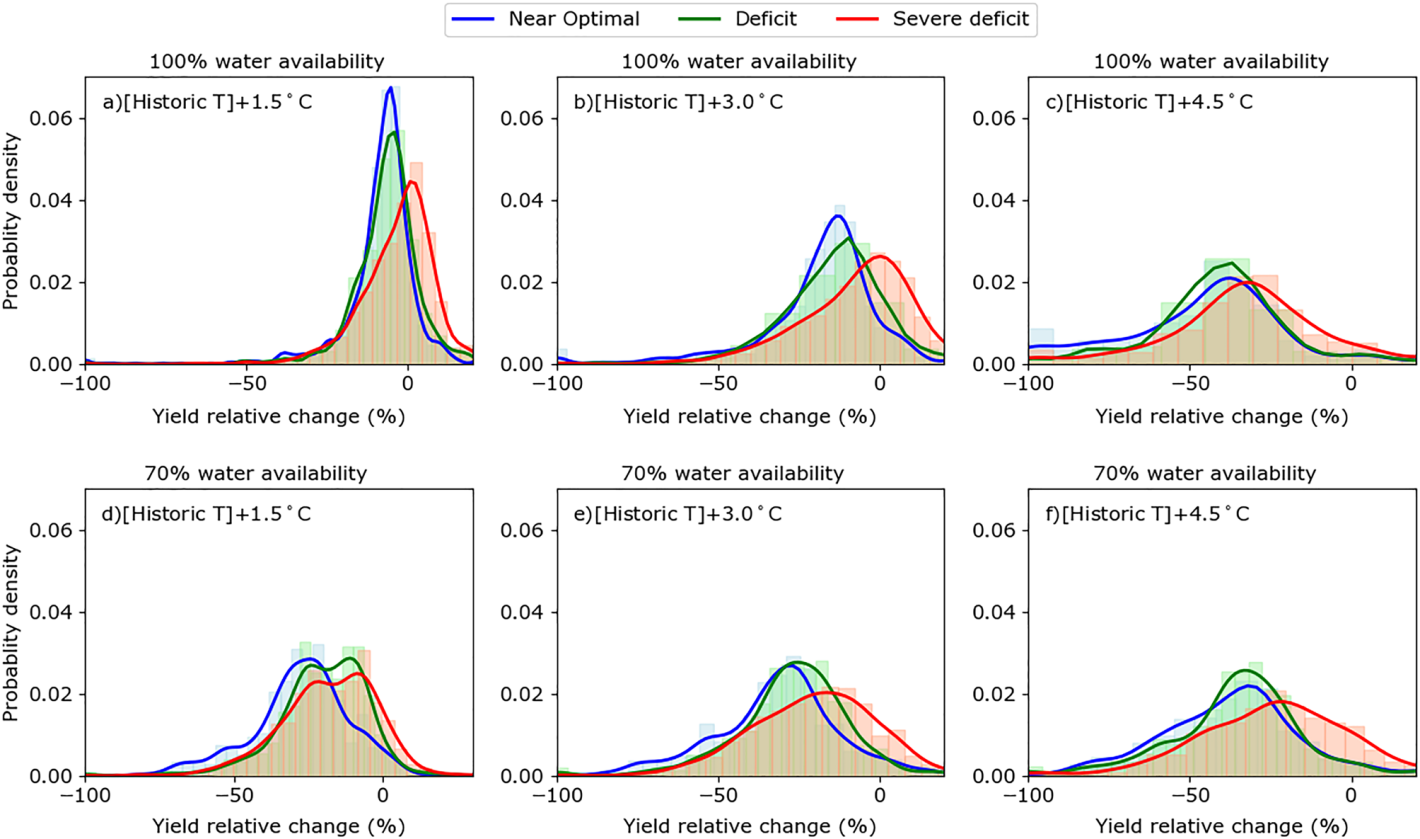
Histograms and probability density function showing the relative change in simulated maize yields under temperature increase scenarios as compared historic yields (baseline years of 1990–2018). The yield relative change is compared under Severe deficit, Deficit, and Near optimal irrigation strategies. Temperature increase scenarios include historic temperature [Historic T] + 1.5 °C, + 3 °C, and + 4.5 °C. The water availability scenarios include full historic water availability (100% water availability) and reduced historic water availability (70% water availability).

Warmer temperatures across different irrigation strategies decreased yield to the greatest extent under the Near optimal irrigation strategy followed by Deficit and Severe deficit irrigation strategies (Fig. 6a–c). An examination of the probability density functions showing the relative yield change for different scenarios of temperature increase (+ 1.5, + 3.0, 4.5 °C) under different irrigation strategies indicated higher reductions under Near optimal and Deficit irrigation strategies compared with Severe Deficit. This shift of probability density function to left signified higher probabilities of yield reductions with increasing temperature (Fig. 6a–c). Compared with the + 1.5 °C scenario, the + 4.5 °C scenario flattened the curve of the distribution function and shifted it toward increased probability of yield reduction. The impact of temperature increase on yield reduction was more apparent when combined with reduced water availability scenarios (70% water availability in Fig. 6d–f). Similar to 100% water availability, a shift toward the left i.e. large reduction was most pronounced for the Near optimal and Deficit irrigation strategies as compared with the Severe Deficit strategy.

**Figure 7:**
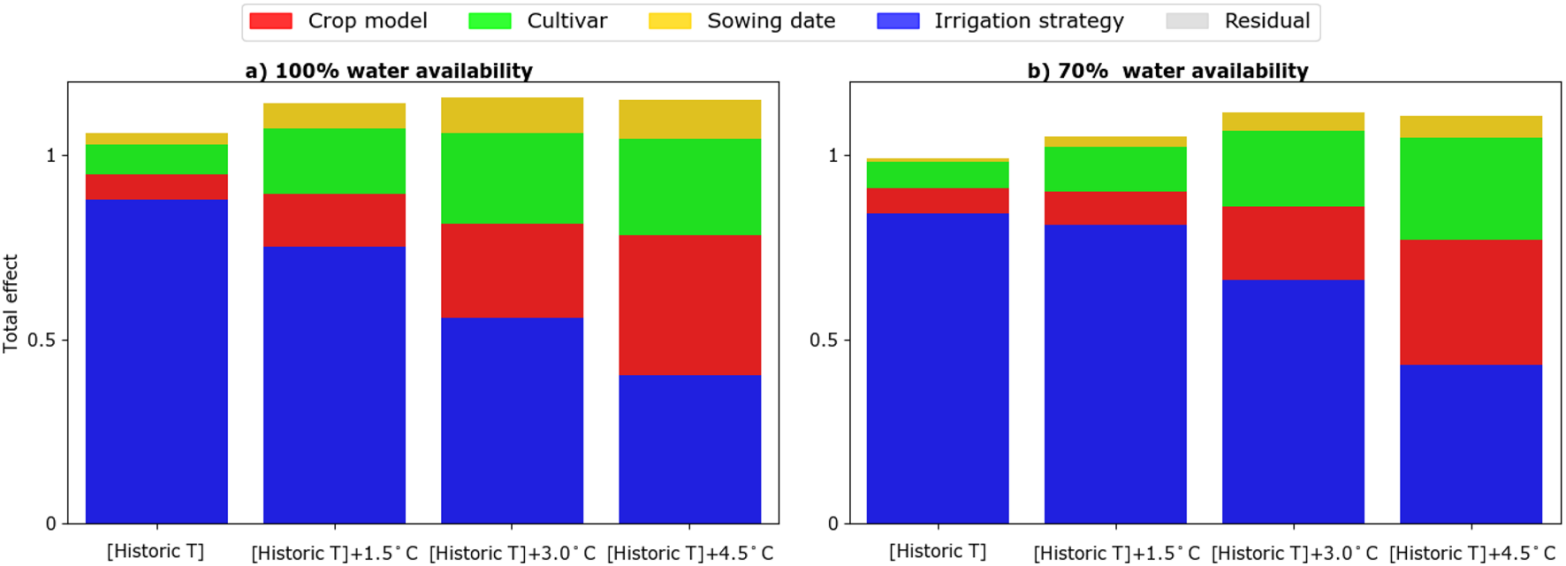
The total effect values of different factors explaining simulated yield under historic temperature ([Historic T]) and three scenarios of temperature increase ([Historic T] + 1.5 °C, [Historic T] + 3 °C, and [Historic T] + 4.5 °C) for: (**a**) 100% water availability and (**b**) 70% water availability.

Decomposing the sources of uncertainty in the simulated yields for the temperature increase and reduced water availability scenarios showed that, in general, irrigation strategy contributed most to the uncertainty. However, this contribution decreased from 80% in the historical period to less than 40% when temperature increased by up to + 4.5 °C (Fig. 7a). As the temperature increased, the relative contribution of choices of crop model and cultivar to uncertainty in simulated yields increased. Comparing the scenarios of decreased water availability indicated that in general all factors contributing to yield uncertainty remained unchanged (Fig. 7b). The total effects for the scenarios of temperature increase showed that as temperature increases, the interaction of cultivar and crop model to total uncertainty becomes notable (Fig. S6a,b).

## Discussion

This paper explored which factor or combination of factors (irrigation strategies, cultivar, sowing date, and crop model) can better explain the variability of observed irrigated maize yield and improve their simulation at a regional scale. Given the fact that irrigated agriculture accounts for the largest share of consumptive water use^42^, the skillness of crop models in simulating regional scale irrigated yields is imperative to assess trade-offs in water allocation across sectors. To date, due to lack of observed data at the regional scale, most studies on simulating irrigated agricultural systems simplified real situations by aggregating rainfed and full irrigation simulations based on the share of production areas^3,17,43,44^. However, yield simulation under full irrigation is not representative of actual condition because it does not consider deficit irrigation or farmer decisions around water allocation among crops. The absence of reliable data on irrigation amount and timing influence greatly the degree of match between observed and simulated yields^45^. Such drawbacks calls for robust simulations of irrigated systems by including different irrigation strategies in combination with influence from other factors like cultivar or sowing date.

Our initial hypothesis was that the mismatch between simulated and observed irrigated yields is related mainly to the missing information on how much and when irrigation water is applied in practice. Combining the simulated maize yields from three irrigation strategies using a linear regression model substantially improved the agreement between simulated and historical yield values (adjusted R^2^ increased from 0.36 in near optimal to 0.56 in Comb8). Since the farmers’ behaviours in relation to irrigation management are not homogeneous, considering one single irrigation strategy as the representative strategy for one whole region reduces model accuracy in terms of yield simulation. In the Mediterranean irrigation schemes, a high variability in irrigation management has been already recorded^19,21^ and such variable behaviours might be even more often seen during extreme years when applying near optimal scheme is not be possible for all farmers. Considering the fact that extreme events will be more frequent under climate warming, it is more critical that our simulations benefit from combination of all implemented irrigation strategies.

It should be noted that the linear combinations of simulated yields under different irrigation strategies in our analysis through improved the results notably, did not cover full uncertainty. Due to lack of yearly farm level data on areas under cultivation of each irrigation strategy and the corresponding yields, we combined variability from different strategies using a regression model. This results in some levels of uncertainty in the regression coefficients obtained from the linear model (Comb8 in Fig. 3) which should be considered in the interpretation of the values. Despite this drawback which likely influenced the results, we still noticed notable improvement after combining three irrigation strategies in Comb8 compared to simulations based on one individual irrigation strategy (Comb 1–3).Although other factors such as cultivar, crop models, or sowing dates further improved simulation results, the highest level of improvement obtained after including irrigation strategies. It highlights the promise of improving crop models' capability in simulating irrigated yields by more regularly measuring and benefiting data on irrigation strategies. Our analysis of the factor decomposition under historical climate conditions offered insights into the contribution of different factors (irrigation strategies, cultivar, sowing date, and crop model) to the uncertainty in simulated yields of irrigated systems. We found that the farmers’ irrigation strategy overweighed other factors explaining yield variability and could already explain over 60% of variability. Previous studies have highlighted that the allocated irrigation water was the determining factor in the region^46,47^ and significantly influenced other management strategies of farmers. For example, when water supply was low, the farmers tried to maximize the net income by change in crop patterns and the amount of water allocated to different crops^22^. However, such detailed levels of data are usually not recorded or available to be integrated into the model. Therefore, there is an urgent need to collect detailed data on irrigation strategies and the time and amount of water allocation to each crop. Several publications have emphasized the concerns associated with data sources in crop modeling^48^. However, no studies specifically quantified the relative contribution of information shortage on irrigation strategies as compared to other factors.

We also found that depending on the applied irrigation strategy, the relative attribution of cultivar, sowing date, and crop model to yield uncertainty differed. For example, during years with high water allocation, information on cultivar became a critical factor for capturing the dynamics of simulated yield. It means that as the crop receives adequate water and does not suffer water stress during its growth period, the length of the vegetative and reproductive period (defined as cultivar type in this study) determines yield variability. The interaction of cultivar with other management factors have been already highlighted in other studies^15^, however, literature lacks information on the relative contribution of this factor under variety of irrigation strategies.

On the other hand, the importance of a correct selection of sowing date is more remarkable with severe deficit irrigation strategy which may be due to a shift in the growth period. Such prioritizing of key factors under different irrigation strategies is paramount because by providing information on one factor, we can find the trade-off for finding the right approach to reduce the uncertainty in simulated yields and eventually increase the reliability of simulated yield for impact assessment studies. In our Andalusia case study, where over 60% of farmers apply deficit irrigation^22^, the remarkable role of cultivar suggests that future studies should include the role of cultivars and collect regular data collection on the type of cultivar practiced in the field to reduce the uncertainty in modeling. Very recently, Ojeda et al.^49^ decomposed the relative contribution of sowing date from other management factors to uncertainty in simulated potato in Tasmania. However, the study did not provide information under each irrigation strategy due to lacks of iter-seasonal information on amount of allocated water to the crops.

We also investigated how the contribution of irrigation strategy, cultivar, sowing date, and crop model factors to uncertainty in simulated yields changes under hypothetical warmer temperatures and reduced water availability scenarios. We increased temperature by up to 4.5 °C ([historic T] + 1.5, 3.0, and 4.5 °C), which is consistent with global temperature increase by up to 5.5 °C predicted in future climate scenarios^50–52^. We found that the factor constellation causing variability in simulated yields changes depending on the degree of increase in temperature. Unlike historic conditions where irrigation strategies were the most determining factor, as temperature increased, our results attested to the significance of other factors (cultivar and crop model). In fact, the importance of the two latter factors rise to the same magnitude as the irrigation strategy. It means that under elevated temperature, irrigation regimes will not be the only important factor driving yield variability.

Our analysis benefited from simulated yields under three types of cultivar, irrigation strategy, and sowing date using three crop models. These models share three phenological stages in common which on one hand enabled us to similarly calibrate them. On the other hand, the selected models (despite some similarity in some phenological stages) differ largely in terms of process representation and model structure and cover a wide range from low (SIMPLACE) to high (APSIM) complexity in the structure. This allowed us to capture the potential variability in yields (i.e. model uncertainty) and can be aligned with the studies of Asseng, et al.^53^ and Wallach, et al.^54^ in which they suggested that depending on the level of increase in temperature and CO_2_ concentration for impact assessment, with three to five models one can already obtains acceptable potential improvement from adding more models.

We noticed that the contribution of crop model (as a factor) to yield variability was smaller than irrigation strategy during the historical period, but increased under high temperature scenarios. The increasing contribution of ‘crop model’ to yield uncertainty with increasing temperature showed that crop models may deviate from each other under extreme climatic conditions which corraborates the earlier findings for increased disagreement between crop models under elevated temperature regimes^53^. Literature emphasized on the exising uncertainty in crop model simulations in a variety of context such as input data^55^, soil condition^56^, or spatial aggregation^57^ and also compared the differences in responses of crop models to water availability^56^ in single-crop water response^24,58^. However, there has been little emphasis on comparing crop model responses in irrigated cropping systems under different irrigation strategies, water allocation levels, and increased temperature scenarios^16^. Evidence for fine model performances concerning water availability is scattered, and the representation of physical processes that interact with crop water use is integrated quite differently in current crop models^24,59^ and can be calculated based on soil water content deficit or transpiration deficit^24,59^. It calls for further research on model comparison in irrigated systems considering additional.

Next to crop models and irrigation strategies, cultivar choice played a decisive role in explaining uncertainty in simulated yields. High temperature speeds up growth development and shortens the growing season due to faster accumulation of thermal time. It results in earlier flowering time and shifts the critical period for yield determination to a different time of the growth season. Therefore, cultivars from long maturity groups with early flowering and late maturity can be more beneficial under temperature increase scenarios. The benefit of using long-cycle cultivars to maintain productivity under increased temperature has been demonstrated in several studies^60,61^. Beside, providing information on cultivar can assist models to more accurately simulate phenological response of crops. However, most of these studies only looked at cultivar effects under rainfed and full irrigation without considering intermediate irrigation strategies as a factor^62^. We explored the interaction of cultivar choice with irrigation streategies to uncertainty in simulated yield under different irrigation strategies and different levels of temperature increase. It is noteworthy that the yield simulations under hypothetical climate change scenarios (increased temperature) were based on the same cultivars we used for simulation of historic period. Considering cultivars which might be tolerant to high temperature, drought-adaptive cultivars and accordingly modifying the phenological characteristics^63^ in simulations may restore the prominence of irrigation strategy to same magnitude as historic period. Parent et al.^64^ also emphasized on the importance of reflecting the crop cycle duration, length of vegetative and reproductive phases for maize under climate change. However, information on the development growth of such cultivars requires more data at the experimental level which were not available for this study.

Compared to the other factors, the contribution of sowing date to yield variability was less apparent under increased temperature scenarios. One possible reason was related to the fact that in our hypothetical temperature increase scenarios ([historic T] + 1.5, 3.0, and 4.5 °C), we assumed a constant daily increase of temperature throughout the year; therefore, any change in sowing date did not influence simulated yield substantially. We also used the three sowing dates observed in historical periods for future scenarios. However, long-term assessments showed that farmers changed the sowing dates in response to climate warming^65,66^. Therefore, we expected the contribution of irrigation strategy on yield variability would again increase when introducing adaptive sowing dates. Nevertheless, testing such hypothesis requires further analysis and was beyond the scope of this paper.

## Conclusion

This paper has implications for historical simulation of irrigated systems and can be used as a protocol for future impact assessment of climate change. Our results give an insight into the role of different factors in improving yield simulations of irrigated maize yield. We here mainly focused on management data as factors and their contribution to yield variability using different models. However, the contribution of other factors can also be included in future studies. For instance, we did not consider the influence of atmospheric CO_2_ concentration under temperature increase scenario, or irrigation infrastructure, or temporal-spatial variability in soil properties^67,68^ that might have changed over the simulated period. Additionally, the biophysical models require more investigation on the processes related to the impact of heat stress on crop failure^25^. It will influence the degree of agreement between simulated and observed yield.. Last but not least, due to the lack of yield data at the farm level, our analysis lacks details on the spatial dimension. Future studies could benefit from retrieving this information from satellite data and explore the drivers of yield variability at spatial dimension.

## Supplementary Information

Supplementary Information.
