# Supplementary material for: Uncertainty in climate change impact studies for irrigated maize cropping systems in southern Spain
Source: Sci Rep. 2022 Mar 8;12:4049. doi: 10.1038/s41598-022-08056-9 (PMC8904498; doi:10.1038/s41598-022-08056-9)
Supplement: Supplementary file 1 — Supplementary Information. [file 41598_2022_8056_MOESM1_ESM.docx]

**Appendix-1**

**APSIM**

We used the APSIM-Maize model within APSIM Next Generation^1^. The model has been implemented in the Plant Modelling Framework and previously described in https://apsimnextgeneration.netlify.app/ and tested across a large range of environments^2,3^. The framework represents the response of phenological and physiological processes (e.g. leaf expansion and biomass accumulation) of a crop to climate and soil inputs and crop management practices. A complete description of the model structure and parameters was provided by Brown, et al. ^4^ and Brown, et al. ^5^. The model contains algorithms that simulate crop phenology, growth, and soil-plant N dynamics. Crop phenology is simulated using thermal time thresholds for each phenological stage. When water and nitrogen conditions are optimum, crop growth rate on a daily step is only limited by solar radiation and is calculated as the product of intercepted solar radiation and radiation use efficiency. When the crop is under water stress, biomass accumulation is calculated as the product of potential crop transpiration (limited by soil moisture, root extent and water uptake capacity) and transpiration efficiency, adjusted for atmospheric vapor pressure deficit^5,6^. Table S2 shows more details on key embedded processes in the models.

**MONICA**

The MONICA (Model for Nitrogen and Carbon dynamics in Agro-ecosystems) model is a process-based agro-ecosystem simulation model for simulating crop growth, water dynamics, and nitrogen dynamics for practical applications. It is an extended version of HERMES ^7^ for the [carbon cycle](https://www.sciencedirect.com/topics/agricultural-and-biological-sciences/carbon-cycle) in soil and plants. The model’s code is generic, meaning that soil‒plant‒water processes can be described using crop-, species-, and soil-specific parameters. MONICA has been tested for a variety of crops in Central Europe^8,9^, and its generic structure can accommodate different crops with different characteristics. Maize growth processes are simulated by dividing growth into seven phenological stages, from emergence to senescence (Table S3). The transition from one stage to another is driven by the accumulation of temperature sum above a base temperature. A capacity approach describes water transport in the soil according to Wegehenkel ^10^. Evapotranspiration is calculated using the Penman-Monteith method^11^ and crop-specific potential evapotranspiration is computed using crop-specific factors during the growing season. Table S2 shows details on main embedded processes in the models.

**SIMPLACE**

The SIMPLACE crop modelling framework ([www.simplace.net/](https://www.simplace.net/)) consists of the Lintul-5 crop growth model^12^ coupled with a modified version of SlimWater^13^. SIMPLACE is coupled with a heat stress module^14^ which is driven by simulated crop canopy temperature^15^. The model has been widely applied for climate change impact assessments for Europe^16-18^. The model explicitly controls for radiation capture, development rates, drought and heat stresses and their interaction. Crop development is a function of temperature accumulation for sowing to emergence, from emergence to flowering and from flowering to maturity. The leaf expansion is calculation based on radiation and converted to biomass using radiation use efficiency. Water stress, daily mean temperature and atmospheric CO_2_ concentration modify radiation use efficiency. Heat stress acts to reduce the grain yield when simulated temperature is greater than a crop specific threshold in the time around anthesis. Heat stress is estimated using simulated hourly crop canopy temperature, which allows accounting for the interaction of crop water status and heat stress impacts^19-21^. The calculated heat stress reduces grain yield around flowering. Table S2 represents details on key embedded processes in the models.


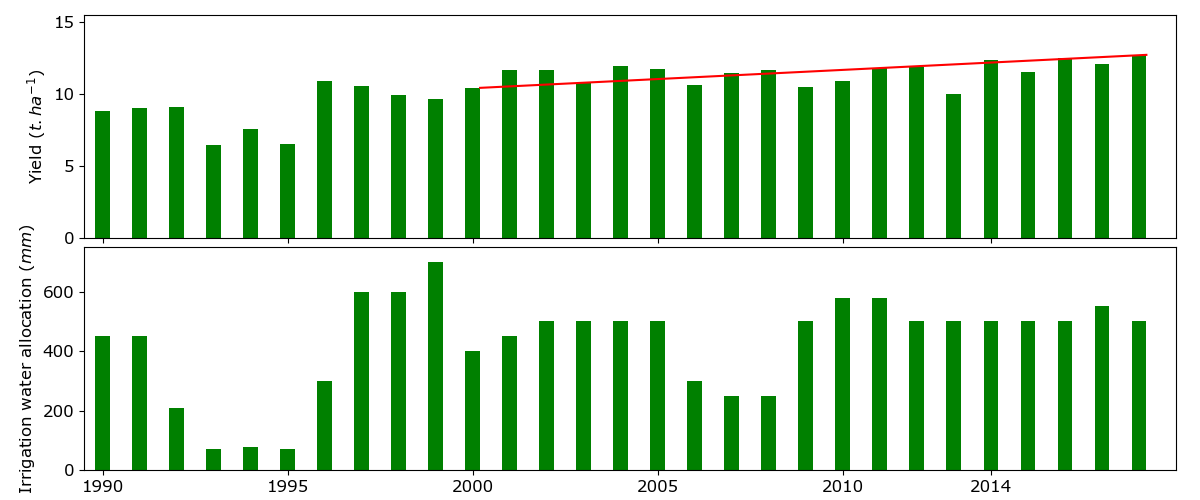


a

b

**Figure S1.** (a) Recorded observed maize yield (t ha^-1^) in Andalusia at NUTS2 level. An upward trend in yield was seen after 2000 and the yield was de-trended during 2000-2018. (b) Recorded water allocation showing the dynamics of available water during the historical period.


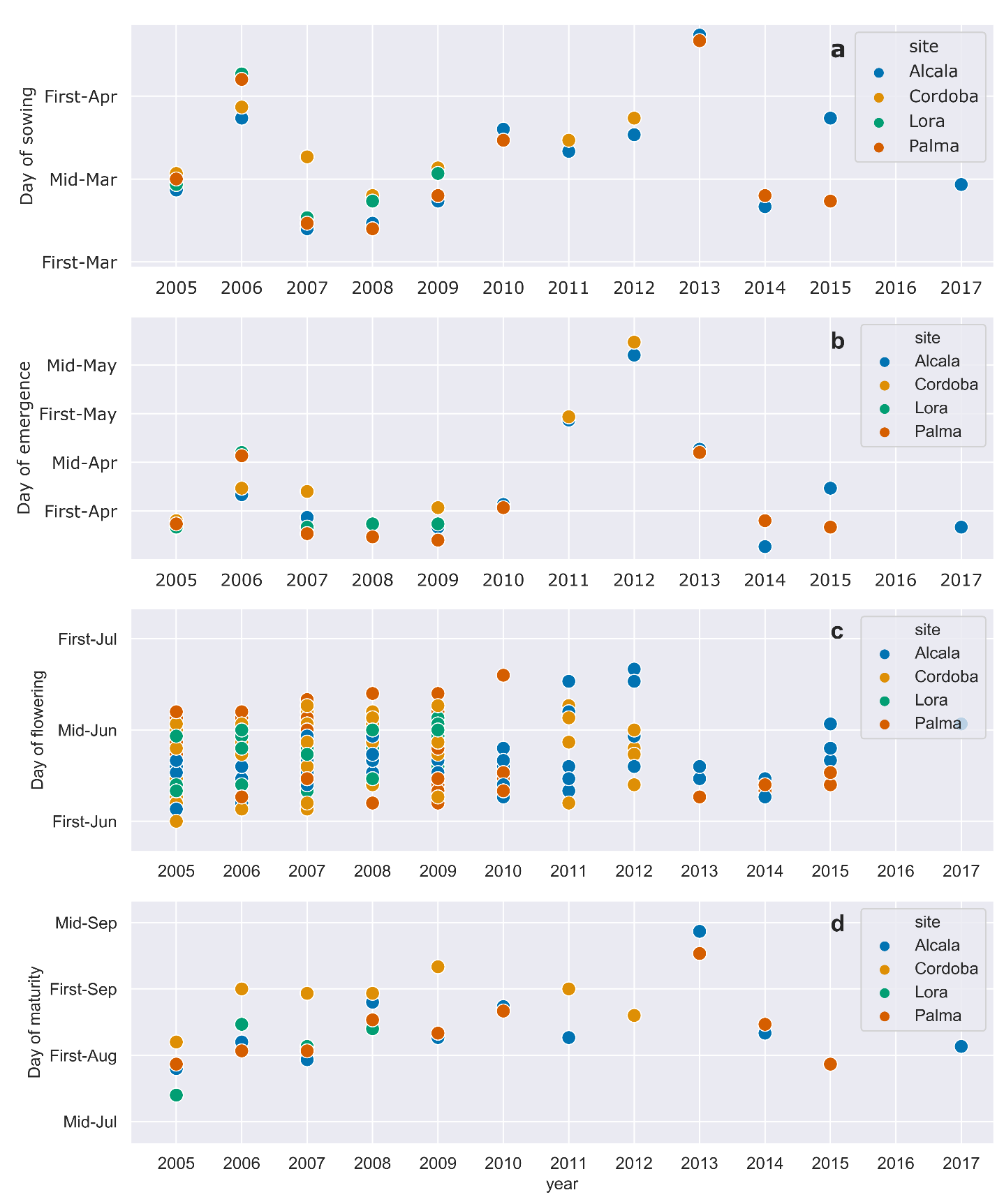


**Figure S2.** Day of year for different phenological stages in Alcala, Cordoba, Lora, Palma during 2005-2017 for a) day of sowing, b) day of emergence, c) day of flowering and d) day of maturity. *Note:* day of flowering was recorded for different cultivars in each site.

**
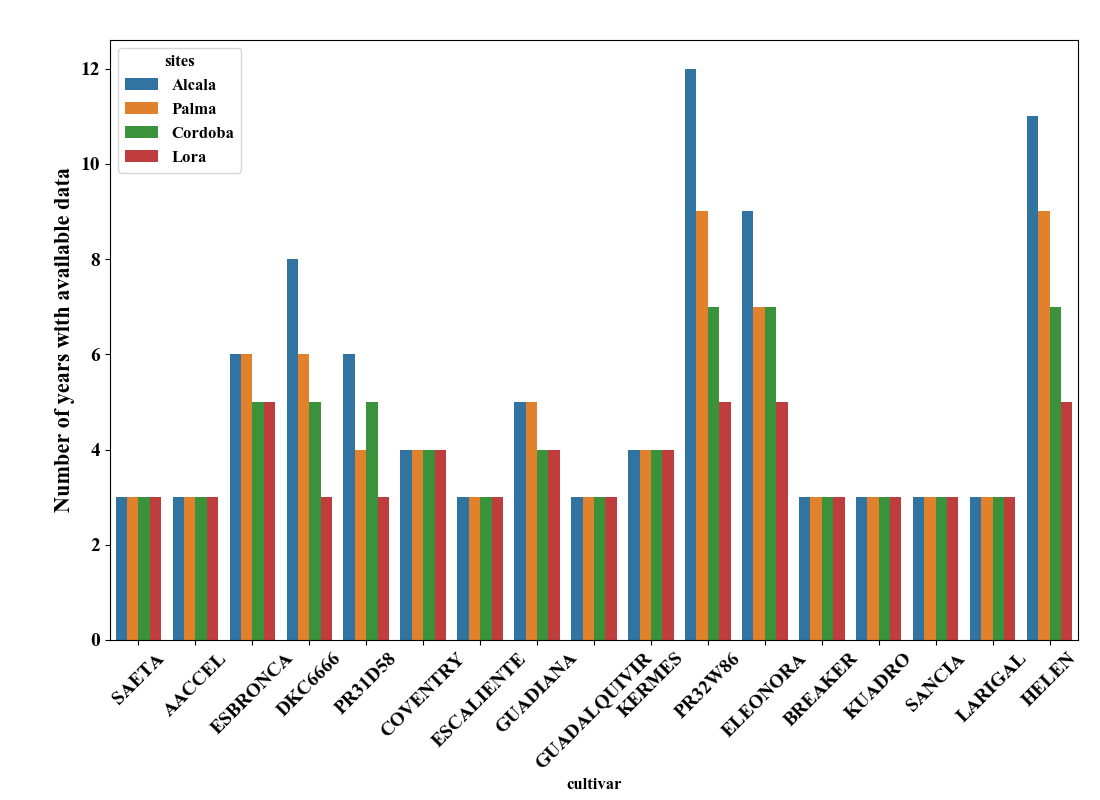
**

**Figure S3.** The 17 selected cultivars (out of 70) used for this study. The y-axis shows the number of years (out of 12 during 2005-2017) for which the data are available per site. Only cultivars whose yield data were available for at least three years in each experimental site were selected.


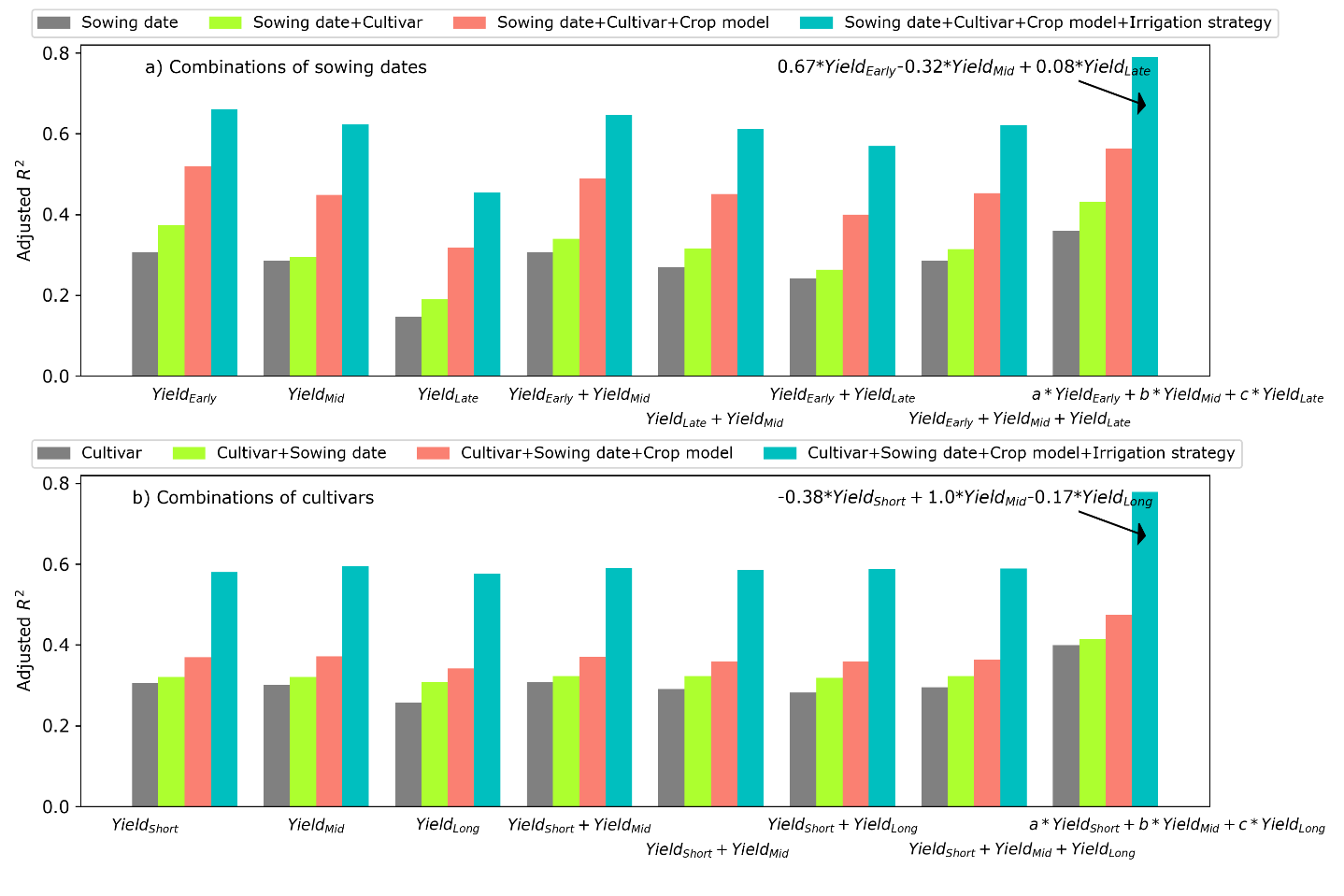


**Figure S4.** The adjusted coefficient of determination (*R^2^*) indicating the degree of agreement between recorded maize grain yield at regional-level in Andalusia and simulated maize yield calculated based on different combinations of a) sowing dates with cultivar, crop model, and irrigation strategy; b) cultivars with other factors as sowing date, crop model, and irrigation strategy during 1990-2018.


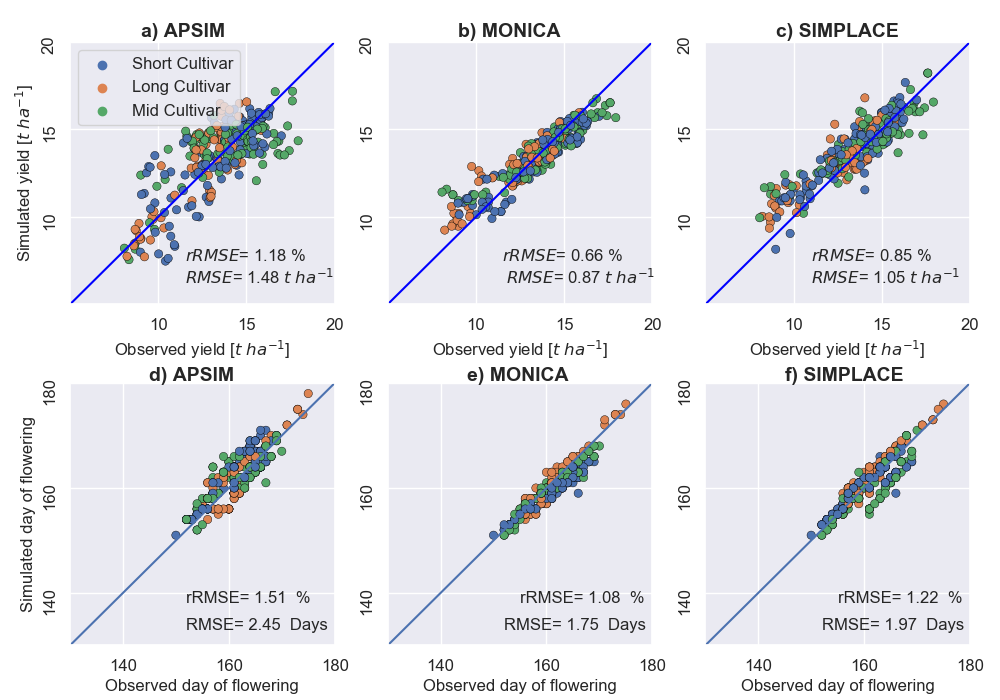


**Figure S5.** (a-c) Comparison between simulated and observed yields in four experimental sites at cultivar level in APSIM, MONICA and SIMPLACE; (d-f) Comparison between simulated and observed day of flowering in four experimental sites at cultivar level in APSIM, MONICA, and SIMPLACE.


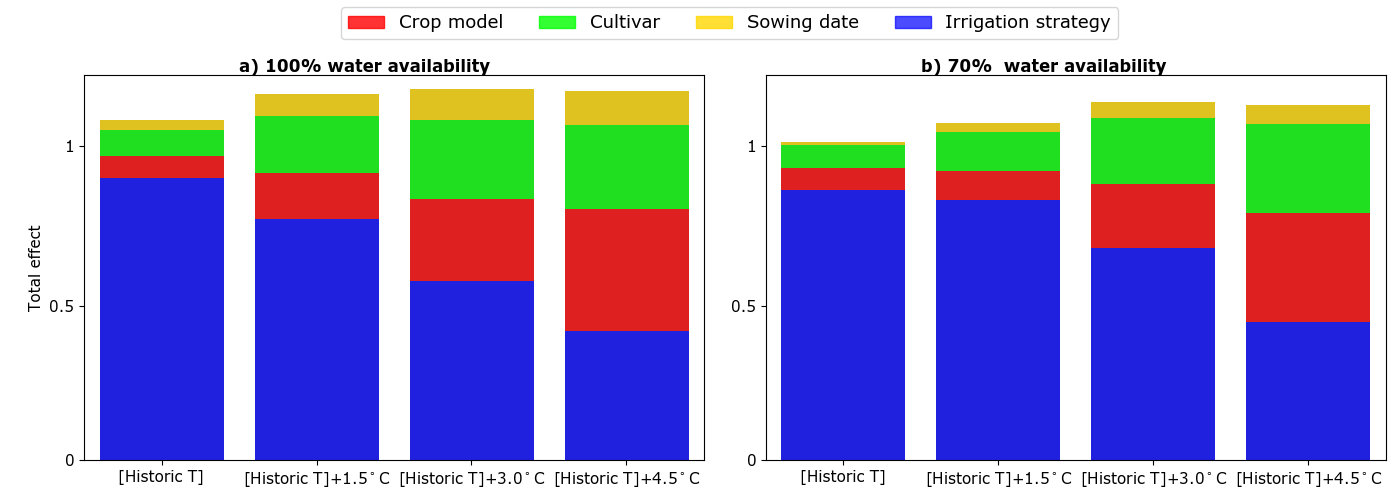


**Figure S6.** The main effect values of different factors explaining simulated yield under historic temperature ([Historic T]) and three scenarios of temperature increase ([Historic T]+1.5°C, [Historic T]+3°C, and [Historic T]+4.5°C) for: a) 100% water availability and b) 70% water availability.

**Table S1.** Characteristics of soil data in experiment and farm sites.

| depth | Silt | Sand | Clay | OM | BD | Wilting point | Field capacity | Pore Volume | KS |
| --- | --- | --- | --- | --- | --- | --- | --- | --- | --- |
| mm |  |  |  | % | g cm^-3^ | mm^3^ mm^-3^ | mm^3^ mm^-3^ | mm^3^ mm^-3^ | mm d^-1^ |
| 0-5 | 0.36 | 0.35 | 0.30 | 3.44 | 1.36 | 0.196 | 0.338 | 0.486 | 20 |
| 5-15 | 0.36 | 0.35 | 0.30 | 2.92 | 1.36 | 0.193 | 0.334 | 0.475 | 20 |
| 15-30 | 0.37 | 0.33 | 0.31 | 2.41 | 1.36 | 0.196 | 0.337 | 0.469 | 20 |
| 30-60 | 0.36 | 0.32 | 0.32 | 2.58 | 1.36 | 0.202 | 0.344 | 0.474 | 20 |
| 60-100 | 0.37 | 0.32 | 0.32 | 1.20 | 1.36 | 0.197 | 0.335 | 0.448 | 20 |
| 100-200 | 0.36 | 0.31 | 0.29 | 1.03 | 1.36 | 0.179 | 0.322 | 0.441 | 20 |

OM: Organic matter

BD: Bulk density

KS: Hydraulic conductivity

**Table S2.** Description of the crop models and water limitation effects.

|  | **SIMPLACE** | **MONICA** | **APSIM** |
| --- | --- | --- | --- |
| **Temperature** | Elevated CO_2_ reduces stomatal conductance in non‐water limited conditions which reduces canopy cooling for any given level of transpiration which results in higher canopy temperature (T )^1^ | None | None |
| **Transpiration** | An empirical function reduces potential transpiration rates with elevated CO_2_ | Crop stomata resistance in FAO‐56 model is calculated according to Yu et al.^7^ depending on CO_2_ concentration, daily gross assimilation, and vapor pressure deficit. | Transpiration efficiency is derived from a *transpiration_efficiency_coefficient* and the vapour pressure deficit estimated from daily temperatures. |
| **RUE** | An empirical function increases RUE with elevated CO_2_ | For photosynthesis of C3 crops, the dependency of maximum Photosynthesis rate and light use efficiency to CO_2_ are described by non‐linear functions proposed by Mitchell et al.^10^ | RUE is a constant value (2) and this parameter is multiplied by adjustment factors to account for the effects of water, temperature, N, vapour pressure deficit and atmospheric carbon dioxide concentration stresses ^22^ |
| **ET Method** | FAO‐56 Penman‐ Monteith^6^ | FAO‐56 Penman‐ Monteith^6^ | FAO‐56 Penman‐ Monteith^6^ adapted by ^23^ |
| **Soil** **water stress** | Soil water stress calculated as ratio of actual to potential transpiration. Used to reduce rates of biomass accumulation and leaf area expansion. Also increase partitioning of biomass to roots. | Soil water stress of the crop is calculated as the relation of actual to potential transpiration, a reduction factor that acts directly specific thresholds are exceeded. | Three water deficit factors are calculated which correspond to four plant processes each having different sensitivity to water stress i.e. photosynthesis, phenology, and leaf-expansion. A water availability ratio is calculated by dividing actual soil water supply by the potential soil water supply. This ratio is used in the relationships illustrated to derive the stress factors for photosynthesis and leaf expansion. |
| **Evaporation/Transpiration distribution** | Dual crop coefficient approach of FAO‐56 | Plant ground coverage determines to what extent transpiration contributes to total evapotranspiration | Potential evapotranspiration is calculated based on the APSIM – MICROMET module ^23^ |

**Table S3.** Description of the crop phenological stages in the three crop models (APSIM, MONICA and SIMPLACE) used in this study.

|  | APSIM | MONICA | SIMPLACE |
| --- | --- | --- | --- |
| **Sowing to Emergence** | sowing to germination  germination to emergence | sowing to emergence | sowing to emergence |
| **Emergence to Flowering** | emergence to end juvenile | emergence to shooting | no intermediate  stages |
|  | end juvenile to floral initiation | shooting to tasseling |  |
|  | floral initiation to flag leaf | tasseling to flowering |  |
|  | flag leaf to flowering |  |  |
| **Flowering to Maturity** | flowering to start grain fill | flowering to corn filling | no intermediate stages |
|  | start grain fill to end grain fill | corn filling |  |
|  | end grain fill to maturity | senescence |  |

**Table S4.** Linear combination of three different irrigation strategies.

| Linear combination of different sowing dates |
| --- |
| ${simulated yield=Yield}_{Early}$ |
| ${simulated yield=Yield}_{Mid}$ |
| ${simulated yield=Yield}_{Late}$ |
| ${simulated yield= a\times Yield}_{Early}+{b\times Yield}_{Mid}$ |
| $simulated yield= a\times{Yield}_{Mid}+{b\times Yield}_{Late}$ |
| $simulated yield={a\times Yield}_{Early}+ b\times{Yield}_{Late}$ |
| ${simulated yield=(Yield}_{Early}+{Yield}_{Mid}+{Yield}_{Late})/3$ |
| $simulated yield= a\times{Yield}_{Early}+b\times{Yield}_{Mid}+c\times{Yield}_{Late}$ |
| Linear combination of different cultivars |
| ${simulated yield=Yield}_{Short}$ |
| ${simulated yield=Yield}_{Mid}$ |
| ${simulated yield=Yield}_{Long}$ |
| ${simulated yield= a\times Yield}_{Short}+ b\times{Yield}_{Mid}$ |
| $simulated yield={a\times Yield}_{Mid}+ b\times{Yield}_{Long}$ |
| $simulated yield={a\times Yield}_{Short}+{b\times Yield}_{Long}$ |
| ${simulated yield=(Yield}_{Short}+{Yield}_{Mid}+{Yield}_{Long})/3$ |
| $simulated yield= a\times{Yield}_{Short}+b\times{Yield}_{Mid}+c\times{Yield}_{Long}$ |

**Table S5**. Statistical p-values under eight different combinations of irrigation strategy (Comb 1-8) and considering different levels of influential factors (“Irrigation strategy”, “Irrigation strategy + Sowing date”, “Irrigation strategy + Sowing date + Cultivar”, and “Irrigation strategy + Sowing date + Cultivar+ Crop model”)

|  | P-values | | | | | | | |
| --- | --- | --- | --- | --- | --- | --- | --- | --- |
| Combination name | $Comb1$ | $Comb2$ | $Comb3$ | $Comb4$ | $Comb5$ | $Comb6$ | $Comb7$ | $Comb8$ |
| $Irrigation strategy$ | 0.004 | 0.007 | 0.005 | 0.005 | 0.002 | 0.006 | 0.0013 | 0.0076 |
| $Irrigation strategy+Sowing date$ | 0.020 | 0.008 | 0.008 | 0.003 | 0.0012 | 0.008 | 0.001 | 0.008 |
| $Irrigation strategy+Sowing date+Cultivar$ | 0.016 | 0.0004 | 3.7E-05 | 0.0010 | 4.0E-05 | 8.1E-05 | 9.2E-05 | 5.3E-05 |
| $Irrigation strategy+Sowing date+Cultivar+Crop model$ | 0.005 | 1.3E-05 | 3.1E-06 | 8.0E-05 | 4.8E-06 | 3.7E-06 | 4.6E-06 | 2.8E-05 |
|  | $Comb1$ | $Comb2$ | $Comb3$ | $Comb4$ | $Comb5$ | $Comb6$ | $Comb7$ | $Comb8$ |
| $Irrigation strategy$ | 0.16 | 0.17 | 0.21 | 0.17 | 0.18 | 0.19 | 0.18 | 0.81 |
| $Irrigation strategy+Sowing date$ | 0.15 | 0.17 | 0.22 | 0.16 | 0.19 | 0.20 | 0.18 | 0.81 |
| $Irrigation strategy+Sowing date+Cultivar$ | 0.19 | 0.22 | 0.31 | 0.21 | 0.25 | 0.27 | 0.24 | 0.84 |
| $Irrigation strategy+Sowing date+Cultivar+Crop model$ | 0.21 | 0.24 | 0.34 | 0.22 | 0.27 | 0.29 | 0.26 | 0.87 |

Reference

1 Holzworth, D. P., Huth, N. I., Devoil, P. G., Zurcher, E. J. *et al.* APSIM - Evolution towards a new generation of agricultural systems simulation. *Environ Modell Softw* **62**, 327-350, doi:10.1016/j.envsoft.2014.07.009 (2014).

2 Hunt, A. G., J. M. , Sharp, P. R., Johnstone & P., S. B. Dividing the risk-theoretical exploration of increasing N management temporal granularity in maize. 377-381 (2019).

3 Morel, J., Parsons, D., Halling, M. A., Kumar, U. *et al.* Challenges for Simulating Growth and Phenology of Silage Maize in a Nordic Climate with APSIM. *Agronomy-Basel* **10**, doi:ARTN 64510.3390/agronomy10050645 (2020).

4 Brown, H. E., Huth, N. I., Holzworth, D. P., Teixeira, E. I. *et al.* Plant Modelling Framework: Software for building and running crop models on the APSIM platform. *Environ Modell Softw* **62**, 385-398, doi:10.1016/j.envsoft.2014.09.005 (2014).

5 Brown, H. E., Huth, N. I., Holzworth, D. P., Teixeira, E. I. *et al.* A generic approach to modelling, allocation and redistribution of biomass to and from plant organs. *In Silico Plants* **1**, doi:ARTN diy00410.1093/insilicoplants/diy004 (2019).

6 Brown, H. E., Teixeira, E. I., Huth, N. I. & Holzworth, D. P. The APSIM Maize Model. (2014).

7 Kersebaum, K. C. Modelling nitrogen dynamics in soil-crop systems with HERMES. *Nutr Cycl Agroecosys* **77**, 39-52, doi:10.1007/s10705-006-9044-8 (2007).

8 Nendel, C., Kersebaum, K. C., Mirschel, W. & Wenkel, K. O. Testing farm management options as climate change adaptation strategies using the MONICA model. *Eur J Agron* **52**, 47-56, doi:10.1016/j.eja.2012.09.005 (2014).

9 Stella, T., Mouratiadou, I., Gaiser, T., Berg-Mohnicke, M. *et al.* Estimating the contribution of crop residues to soil organic carbon conservation. *Environ Res Lett* **14**, doi:ARTN09400810.1088/1748-9326/ab395c (2019).

10 Wegehenkel, M. Test of a modelling system for simulating water balances and plant growth using various different complex approaches. *Ecological Modelling* **129**, 39-64, doi:Doi 10.1016/S0304-3800(00)00221-0 (2000).

11 Allen, R. G., Pereira, L. S., Raes, D. & Smith, M. Crop evapotranspiration. Guidelines for computing crop water requirements. . (Roma, 1998).

12 Wolf, J. LINTUL5: Simple generic model for simulation of crop growth under potential, water limited and nitrogen, phosphorus and potassium limited conditions. (Plant Production Systems Group, Wageningen University, Wageningen, 2012).

13 Addiscott, T. M. & Whitmore, A. P. Simulation of Solute Leaching in Soils of Differing Permeabilities. *Soil Use Manage* **7**, 94-102, doi:DOI 10.1111/j.1475-2743.1991.tb00856.x (1991).

14 Gabaldón-Leal, C., Webber, H., Otegui, M. E., Slafer, G. A. *et al.* Modelling the impact of heat stress on maize yield formation. *Field Crops Research* **198**, 226-237, doi:<http://dx.doi.org/10.1016/j.fcr.2016.08.013> (2016).

15 Webber, H. A., Ewert, F., Kimball, B., Siebert, S. *et al.* Simulating canopy temperature for modelling heat stress in cereals. *Environmental Modelling & Software* **77**, 143-155 (2016).

16 Webber, H., Ewert, F., Olesen, J. E., Müller, C. *et al.* Diverging importance of drought stress for maize and winter wheat in Europe. *Nature Communications* **9**, 4249, doi:10.1038/s41467-018-06525-2 (2018).

17 Zhao, G., Webber, H., Hoffmann, H., Wolf, J. *et al.* The implication of irrigation in climate change impact assessment: a European wide study. *Global Change Biology* **21**, 4031–4048, doi:10.1111/gcb.13008 (2015).

18 Zimmermann, A., Webber, H., Zhao, G., Ewert, F. *et al.* Climate change impacts on crop yields, land use and environment in response to crop sowing dates and thermal time requirements. *Agricultural Systems* **157**, 81-92 (2017).

19 Lobell, D. B., Hammer, G. L., McLean, G., Messina, C. *et al.* The critical role of extreme heat for maize production in the United States. *Nat Clim Change* **3**, 497-501, doi:10.1038/Nclimate1832 (2013).

20 Troy, T. J., Kipgen, C. & Pal, I. The impact of climate extremes and irrigation on US crop yields. *Environ Res Lett* **10**, doi:Artn 05401310.1088/1748-9326/10/5/054013 (2015).

21 Carter, E. K., Melkonian, J., Riha, S. J. & Shaw, S. B. Separating heat stress from moisture stress: analyzing yield response to high temperature in irrigated maize. **11** (2016).

22 Ojeda, J. J., Rezaei, E. E., Remenyi, T. A., Webb, M. A. *et al.* Effects of soil- and climate data aggregation on simulated potato yield and irrigation water requirement. *Science of the Total Environment* **710**, doi:10.1016/j.scitotenv.2019.135589 (2020).

23 Snow, V. O. & Huth, N. I. The APSIM–Micromet module. (Auckland, 2004).
